# Linfoma de células del manto con afectación de vía aérea múltiple tratado mediante resección con broncoscopio rígido. Caso clínico y revisión de la literatura

**DOI:** 10.1016/j.opresp.2022.100164

**Published:** 2022-02-09

**Authors:** María Teresa Tejedor Ortiz, Ricardo García Luján, Gema María Siesto López, Maria Piñeiro Roncal, Fernando Revuelta Salgado, Eduardo de Miguel Poch

**Affiliations:** Servicio de Neumología, Hospital Universitario 12 de Octubre, Madrid, España

*Estimado Editor:*

Presentamos el caso de un varón de 84 años con antecedentes de anemia hemolítica autoinmune en tratamiento con corticoterapia y linfoma de células del manto indolente. Este fue diagnosticado 4 años antes, con afectación nodal (adenopatías mediastínicas, axilares, retroperitoneales, iliacas) y extranodal (bazo y médula ósea). Fue manejado de forma conservadora.

Consultaba por cuadro de una semana de evolución de disnea progresiva hasta hacerse de mínimos esfuerzos, tos seca y dolor pleurítico en hemitórax izquierdo. En la exploración física presentaba estridor inspiratorio. En la analítica sanguínea únicamente destacaban valores elevados de LDH. En la radiografía de tórax (fig. 1a) se observó atelectasia de lóbulo superior izquierdo (LSI) y lóbulo medio (LM), así como una lesión endotraqueal y ensanchamiento mediastínico, por lo que se decidió realizar una tomografía computarizada (TC) torácica (fig. 1b). En ella, se objetivaron varias lesiones endotraqueales y endobronquiales, destacando una lesión endotraqueal de 1,5 cm de tamaño, a 3 cm de la carina traqueal, que ocluía el 75% de la luz traqueal. También presentaba una lesión endobronquial que ocluía completamente el bronquio lobar superior izquierdo y que condicionaba una atelectasia completa de este lóbulo, y adenopatías mediastínicas en todas las estaciones, entre ellas, adenopatías hiliares derechas con atelectasia parcial secundaria del LM.

**Figura 1:**
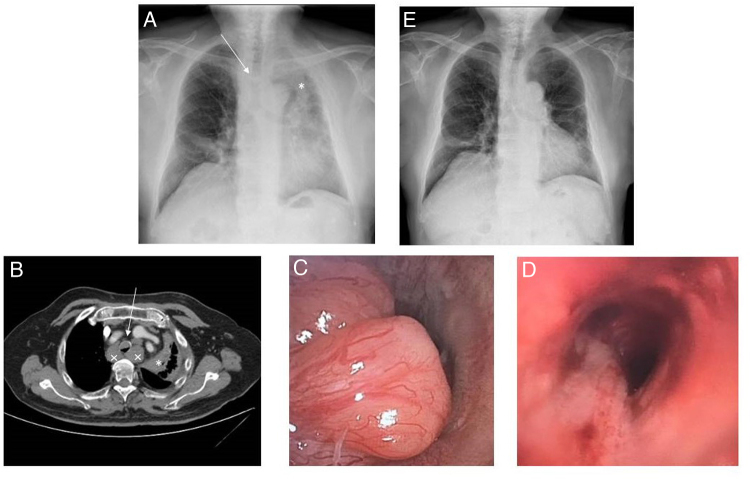
. a. Radiografía de tórax PA previa al procedimiento. Se observa lesión endotraqueal (flecha) y atelectasia de LSI (*). b. TC de tórax, previa al procedimiento, donde se observa una lesión endotraqueal de 1,5 cm (flecha), atelectasia de LSI (*) secundaria a lesión endobronquial y adenopatías mediastínicas (×). c. Fotografía realizada durante la broncoscopia terapéutica, antes de la resección. Se observa una lesión polipoidea endotraqueal, que obstruye más del 80% de la luz traqueal. d. Fotografía realizada durante la broncoscopia terapéutica, después de la resección. Se observa la luz traqueal permeable tras resección de tumoración polipoidea con broncoscopio rígido. e. Radiografía de tórax PA posterior al procedimiento. En ella se observa la resolución de la atelectasia de LSI y la tráquea permeable.

Dada la clínica del paciente y los hallazgos de la TC, se programó broncoscopia en quirófano. Se realizó exploración con broncoscopio rígido, visualizándose una tumoración de aspecto cerebroide a unos 3 cm de la carina traqueal, que obstruía un 80% de la luz traqueal (fig. 1c) y se extendía distalmente, confluyendo con otro implante a 1 cm de la carina traqueal. En el sistema bronquial izquierdo presentaba una lesión similar, que obstruía el LSI e infiltraba la entrada al lóbulo inferior izquierdo. En el sistema bronquial derecho se observó una tumoración que obstruía el LM. Se resecaron con broncoscopio rígido las 2 lesiones traqueales, con mínimo sangrado y quedando una luz normal, dejando únicamente la base de implantación de las mismas (fig. 1d). Seguidamente, con el videobroncoscopio terapéutico y con una pinza flexible se consiguió desobstruir parcialmente tanto el LSI como el LM, quedando una mucosa infiltrativa a nivel de ambos lóbulos, con buena luz distal.

El paciente mejoró clínicamente de manera inmediata con resolución de la disnea y del estridor, y radiológicamente (fig. 1e). El examen histológico mostró infiltración submucosa por proceso linfoproliferativo de bajo grado, compatible con metástasis de su linfoma previo conocido. Se realizaron técnicas inmunohistoquímicas que mostraron una población de células atípicas CD5, CD20, PAX20, SOX11 y ciclina D1 positivas, y CD23 y CD3 negativas, congruente con linfoma de células del manto. Ante el diagnóstico, fue derivado a consultas de Hematología, donde se decidió iniciar tratamiento con rituximab en monoterapia, dada la edad avanzada del paciente, con mejoría, persistiendo tras un año de seguimiento sin lesiones de nueva aparición.

El linfoma de células del manto es un linfoma de células B maduras no Hodgkin, incurable y poco frecuente, que constituye entre el 2 y el 10%^1^ de este tipo de linfomas, y presenta una incidencia de alrededor de 0,5 por cada 100.000 personas, siendo más prevalente en hombres, de raza blanca y de edad avanzada^1^. La translocación (11;14)(q13;q32)^2^ está presente en prácticamente todos los casos. Histopatológicamente, se reconocen 3 patrones de crecimiento en los ganglios linfáticos afectados: difuso, que representa el 61% y típicamente muestra una población linfocitaria de pequeño-mediano tamaño, con un núcleo irregular y un citoplasma escaso, expansión de la zona de manto (26%) o nodular (13%)^1^, ^3^.

Estas células expresan marcadores típicos de células B, como CD19 y CD20. Típicamente son positivas para CD5 y CD43, y negativas para CD3, CD23, CD10, CD11c y bcl6^3^. Además, la mayoría expresan ciclina D1 como resultado de la sobreexpresión de esta molécula inducida por la translocación cromosómica previamente mencionada. Aquellos linfomas de células del manto que no expresan este marcador pueden sobreexpresar ciclina D2, D3 o SOX11^4^, el cual se expresa en el 90%^1^ y, por lo tanto, puede ser de ayuda en el diagnóstico.

Al diagnóstico, un tercio de los pacientes presentan síntomas y más de dos tercios presentan enfermedad diseminada. Es una entidad con afectación frecuente de los ganglios linfáticos, el bazo, el hígado y la médula ósea, y extranodal, especialmente gastrointestinal, siendo muy rara la afectación pulmonar o endobronquial^1^.

En el caso de la afectación pulmonar, existen 3 patrones que se pueden observar en la TC de tórax. El primero y más frecuente es la aparición de nódulos pulmonares de predominio en lóbulos inferiores. El segundo consiste en un patrón reticular central, con distribución peribronquial y perivascular. El tercero y menos frecuente se presenta como un infiltrado alveolar. La afectación endobronquial del linfoma de células del manto suele revelar una mucosa difusamente infiltrada, y más raramente lesiones polipoideas. Esta afectación endobronquial es extremadamente rara, existiendo pocos casos descritos hasta el momento en la literatura^7^, ^8^, ^9^, ^10^, ^11^, ^12^. Independientemente del patrón radiológico, para el diagnóstico definitivo es indispensable una confirmación histológica.

En pacientes de edad avanzada, como es nuestro caso, o en pacientes con un MIPI bajo, se puede decidir un tratamiento conservador y observación^13^. Cuando estos pacientes comiencen con sintomatología, las terapias de primera línea que se pueden utilizar incluyen R-bendamustina o R-CHOP (± mantenimiento con rituximab)^13^.

Nuestro caso clínico tiene datos de especial interés por sus peculiaridades. En primer lugar, la afectación traqueobronquial del linfoma de células del manto es una entidad muy poco frecuente y no suele afectar a tantas localizaciones como en nuestro paciente. Además, la presentación en forma de lesiones polipoideas múltiples, en un paciente diagnosticado ya previamente de un linfoma de células del manto indolente, nodal y extranodal al diagnóstico es excepcional y hasta el momento solo hay algún caso descrito de criorresección, pero no de resecciones endoscópicas con broncoscopia rígida. Este tratamiento, en nuestro caso, que tenía lesiones que obstruían de manera significativa la luz traqueal y bronquial y comprometían la vida del paciente, permitió, por un lado, un tratamiento que mejoró la sintomatología del paciente de manera inmediata, y por otro, la obtención de muestras tisulares de buena calidad y tamaño que permitieron la realización de técnicas inmunohistoquímicas y el diagnóstico histológico de confirmación de metástasis de linfoma de células del manto que justificaron el inicio del tratamiento sistémico.

## Consentimiento informado

El paciente ha otorgado su consentimiento para la publicación de este artículo.

## Financiación

Los autores declaran que no hay ninguna entidad financiadora.

## Conflicto de intereses

Los autores declaran no tener ningún conflicto de intereses.
